# Terpenes in *Cannabis sativa* Inhibit Capsaicin Responses in Rat DRG Neurons via Na^+^/K^+^ ATPase Activation

**DOI:** 10.3390/ijms242216340

**Published:** 2023-11-15

**Authors:** Uma Anand, Praveen Anand, Mikael Hans Sodergren

**Affiliations:** 1 Faculty of Medicine, Imperial College London, Hammersmith Hospital, Du Cane Rd, London W12 ONN, UK; p.anand@imperial.ac.uk (P.A.); m.sodergren@imperial.ac.uk (M.H.S.); 2 Curaleaf International Ltd., 179 Great Portland Street, London W1W 5PL, UK

**Keywords:** chronic pain, terpenes, membrane potential, hyperpolarization, sensory neurons

## Abstract

Terpenes in *Cannabis sativa* exert analgesic effects, but the mechanisms are uncertain. We examined the effects of 10 terpenes on capsaicin responses in an established model of neuronal hypersensitivity. Adult rat DRG neurons cultured with neurotrophic factors NGF and GDNF were loaded with Fura2AM for calcium imaging, and treated with individual terpenes or vehicle for 5 min, followed by 1 µMol capsaicin. In vehicle treated control experiments, capsaicin elicited immediate and sustained calcium influx. Most neurons treated with terpenes responded to capsaicin after 6–8 min. Few neurons showed immediate capsaicin responses that were transient or normal. The delayed responses were found to be due to calcium released from the endoplasmic reticulum, as they were maintained in calcium/magnesium free media, but not after thapsigargin pre-treatment. Terpene inhibition of calcium influx was reversed after washout of medium, in the absence of terpenes, and in the presence of the Na^+^/K^+^ ATPase inhibitor ouabain, but not CB_1_ or CB_2_ receptor antagonists. Thus, terpenes inhibit capsaicin evoked calcium influx by Na^+^/K^+^ ATPase activation. Immunofluorescence showed TRPV1 co-expression with α1β1 Na^+^/K^+^ ATPase in most neurons while others were either TRPV1 or α1β1 Na^+^/K^+^ ATPase positive.

## 1. Introduction

The neuromodulatory effects of cannabinoids have been recognized for millenia in traditional medicine, including for pain relief [1]. Following the opioid crisis, attention has been focussed on developing alternatives including cannabinoid-based pain therapies, as chronic pain remains an unmet need. The best known of the phytocannabinoids is Δ^9^tetrahydrocannabinol (THC), the only known psychoactive component, along with many other cannabinoids with potential therapeutic benefits, such as cannabidiol (CBD), and cannabigerol (CBG) [2]. Amongst the several hundred components in *Cannabis sativa* are terpenes, which are produced in small and varying amounts in different cultivars of *C. sativa*, leading to potential variation in their effects [3]. Some of these, including limonene, phytol, borneol, terpineol, and caryophyllene, provide pain relief via calcium channel inhibition [4]. Similarly, antinociceptive and anti-tumour effects of α-phellandrene were reported, although the mechanisms were unknown [5]. Terpenes as a class of compounds are generally described as safe by the FDA, with low toxicity that extends their efficacy to a variety of indications including chronic pain and anxiety [6].

Behavioural studies in preclinical models of pain have demonstrated antinociceptive effects of terpenes, with camphene and α-bisabolol inhibiting inflammatory and neuropathic pain via Cav3.2 T-type calcium channels in mice [4]. While geraniol, linalool, α-humulene, and β-pinene elicited the tetrad of cannabimimetic behavioural effects in mice, they were only partially reversed by CB_1_ or adenosine antagonists [7], indicating the involvement of a CB_1_ receptor independent mechanism of action. In a rat model of arthritis, local application of 1 and 5 mg/kg sc myrcene reduced joint pain and inflammation via a cannabinoid receptor mechanism [8]. Recent studies have also reported analgesic effects of camphor compared with pregabalin in models of neuropathic pain [9]. In ascertaining a mechanism of action, previous studies described absence of terpene activity at CB_1_ or CB_2_ receptors [10], and Heblinski et al. [11] reported absence of a modulatory effect of terpenes on endocannabinoids or phytocannabinoids in TRPV1 and TRPA1 transfected HEK cells.

In this study, we have examined the potential antinociceptive effects of terpenes in blocking pain signalling in sensory neurons using an established in vitro model of neuronal hypersensitivity. Ten different terpenes were assessed for their effects on calcium influx in sensory neurons, and in modulating their responses to a noxious stimulus using capsaicin, the hot ingredient of chilli peppers.

Capsaicin has highly selective effects on primary afferent C- and Aδ- nerve fibres [12,13], such as membrane depolarization and increased membrane permeability to cations including calcium, sodium, and potassium [14,15,16]. The effects of capsaicin are specifically mediated via activation of the vanilloid receptor 1 (VR1, later renamed as the transient receptor potential vanilloid subtype 1 or TRPV1), expressed in the small nociceptive primary sensory neurons of the dorsal root ganglia (DRG) [17,18]. The expression and sensitivity of TRPV1 is enhanced by the neurotrophic factors NGF and GDNF in rodent [19,20] and human DRG neurons [21].

TRPV1 expression and function has been characterised well as being activated by noxious stimuli such as low pH, temperature of 43 °C and above, inflammatory mediators, and capsaicin, the membrane permeable hot ingredient of chilli peppers [22,23,24]. The TRPV1 receptor is a homo-tetramer with six trans-membrane loops and intracellular N- and C- termini, located in the plasma membrane and endoplasmic reticulum. TRPV1 receptor activation in the plasma membrane leads to Ca^+^ influx and increased cytosolic Ca^+^, while activation of endoplasmic reticulum membrane bound TRPV1 causes Ca^+^ release from intracellular stores [25,26]. Although TRPV1 is a polymodal nociceptor activated by capsaicin, protons and heat, the responses to these stimuli depend on the membrane potential, with depolarized states favouring channel activation, and hyperpolarization having an inhibitory effect [27,28,29,30]. Thus, drug candidates that modulate the membrane potential to hyperpolarized states, can potentially inhibit TRPV1 activation.

In this study we have used an in vitro model of neuronal hypersensitivity, as previously described [31,32], to determine the effects of terpenes, on TRPV1 signalling in rat DRG neurons.

## 2. Results

### 2.1. Calcium Imaging

**Figure 1:**
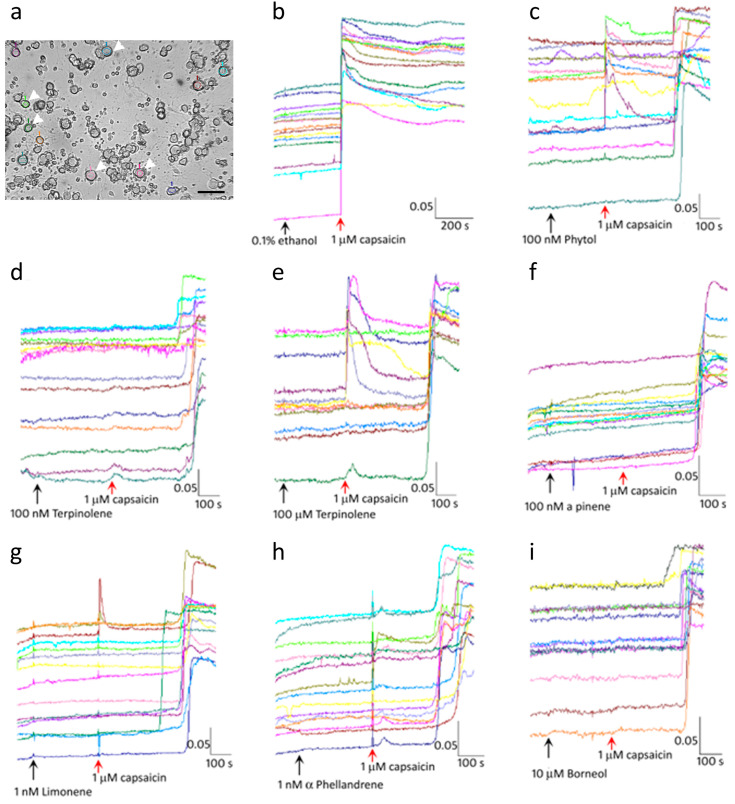
Terpene inhibition of capsaicin responses. Photomicrograph showing a brightfield image of DRG neurons at 10× magnification. Examples of highlighted neurons are indicated by arrowheads. Scale bar = 50 microns (**a**). Up to 15 neurons were identified in each experiment (each neuron being represented by a different colour for recording changes in intracellular calcium) and showed a stable baseline after vehicle application (black arrow). In the presence of vehicle, capsaicin application (red arrow), resulted in an immediate response with increased intracellular calcium, sustained for several minutes (**b**). Few transient capsaicin responses were observed in the presence of 100 nM Phytol, although most were delayed (**c**), 100 nM terpinolene (**d**). Some neurons showed transient responses to capsaicin in the presence of 100 µM terpinolene followed by delayed capsaicin responses (**e**). Similarly, delayed responses were seen in the presence of 100 nM Pinene (**f**), 1 nM limonene (**g**), 1 nM α-Phellandrene (**h**), and 10 µM Borneol (**i**). Scale bar: *X*-axis shows the time in seconds. *Y*-axis shows the intracellular 340/380 (bound/unbound calcium) ratio, in individual neurons depicted by different colours.

Vehicle control: Baseline recording of the 340/380 ratio in individual highlighted neurons (Figure 1a), was obtained for about half a minute. Control neurons were treated with 0.1% ethanol (vehicle) followed 5 min later by 1 µM capsaicin. No change in baseline was observed in the presence of vehicle, and capsaicin sensitive neurons responded immediately, within seconds of application, as an immediate increase in intracellular 340/380 ratio that was sustained for several minutes (Figure 1b).

#### 2.1.1. Response to Terpene Application

After the establishment of a stable baseline, individual terpenes were applied at different concentrations, followed 5 min later by 1 µM capsaicin. None of the terpenes elicited calcium influx, and baseline changes were rarely observed following application of any of the terpenes.

#### 2.1.2. Terpenes Inhibit Capsaicin Activation of TRPV1

**Figure 2:**
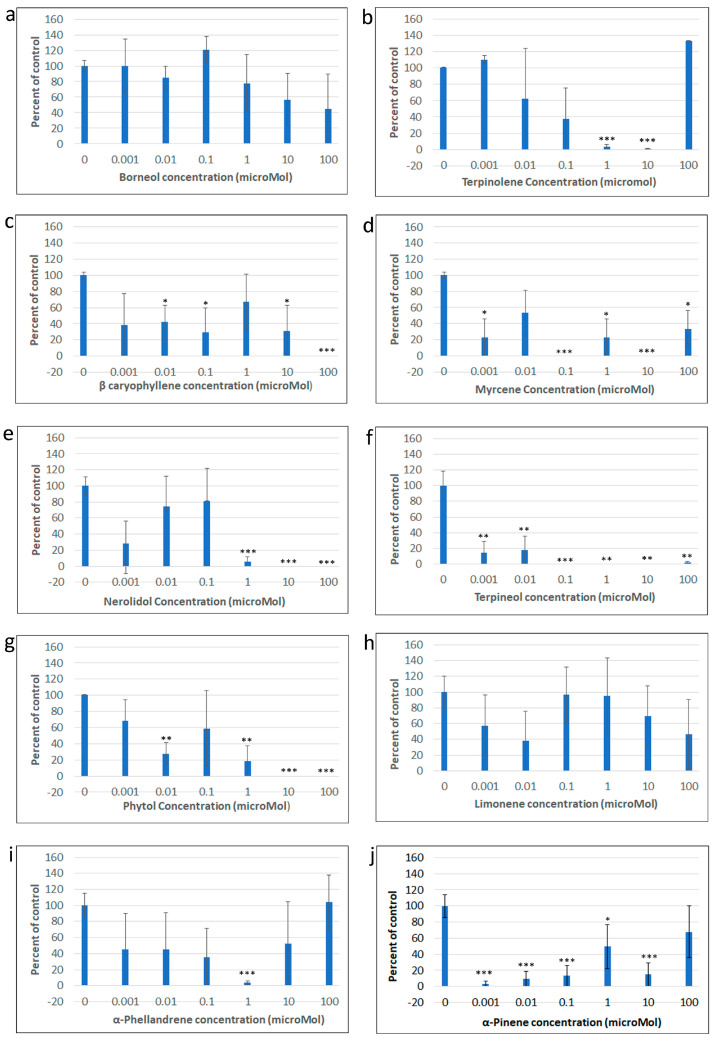
Graphs showing mean ± SEM capsaicin response amplitudes normalised to vehicle treated controls, in the presence of Borneol (**a**) Terpinolene (**b**), β-caryophyllene (**c**), myrcene (**d**), nerolidol (**e**), terpineol (**f**), phytol (**g**), limonene (**h**), α-phellandrene (**i**) and α-pinene (**j**) at different doses (n = 3–4 rats/terpene). Capsaicin response amplitudes were significantly reduced compared to vehicle treated controls as indicated * *p* < 0.05, ** *p* < 0.01, *** *p* < 0.001 Unpaired *T*-test.

In the presence of the terpenes Borneol, Caryophyllene, Limonene, α-Phellandrene, Phytol, α-Pinene, Nerolidol, Terpinolene, Myrcene and α-Terpineol, at concentrations of 0.001, 0.01, 0.1, 1, 10 and 100 µM, capsaicin responses were completely inhibited for 6–8 min, in the majority of capsaicin sensitive neurons, which was not dose related. For 6–8 min after applying capsaicin, the baseline remained unchanged, followed by increased calcium levels in a subset of capsaicin sensitive neurons (Figure 1c–i). Some neurons demonstrated immediate capsaicin responses that were transient, followed by a second increase in calcium after 6–8 min, and very few neurons demonstrated similar responses to vehicle treated controls. At some terpene concentrations, capsaicin response amplitudes were completely inhibited while at others they were reduced (Figure 2). Except for Borneol and limonene, a large proportion of capsaicin sensitive neurons showed complete inhibition in the presence of all terpenes (Table 1).

#### 2.1.3. Terpene Mediated Capsaicin Response Inhibition Is Reversible

**Figure 3:**
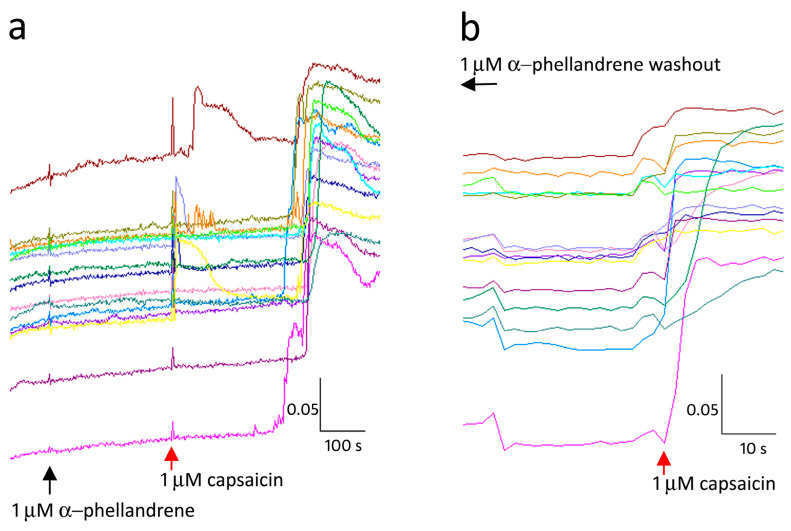
Terpene inhibition of capsaicin responses is reversible. In the presence of α-phellandrene capsaicin responses were inhibited at the point of application (red arrow, (**a**)). Following washout of medium, capsaicin application 45 min later, restored immediate responses in the absence of α- phellandrene (**b**). Scale bar: *X*-axis shows time in seconds and *Y*-axis shows the 340/380 (bound/unbound calcium) ratio, in individual neurons depicted by different colours.

After confirming the TRPV1 inhibitory effects of all the terpenes at different concentrations, we examined the mechanism of action of this effect. Neurons demonstrating delayed capsaicin responses in the presence of 1 µM α-phellandrene (Figure 3), showed immediate and sustained capsaicin responses similar to controls, after washout of medium, and capsaicin application 45 min later, in the absence of α-phellandrene. Similarly, capsaicin response delay due to α- pinene was also reversed after washout.

#### 2.1.4. Terpenes Block Calcium Influx but Not Calcium Release

**Figure 4:**
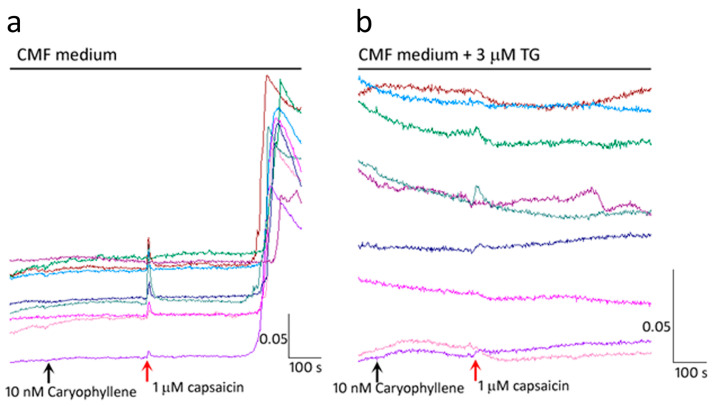
Calcium influx is blocked by β-Caryophyllene. In calcium/magnesium free medium (CMF), capsaicin responses were completely inhibited for 6–8 min in the presence of 10 nM caryophyllene, followed by elevated intracellular calcium (**a**). The delayed capsaicin responses due to 10 nM caryophyllene were eliminated after washout and thapsigargin (TG) treatment, in Ca^+2^/Mg^+^ free medium (**b**). Scale bar: *X*-axis shows time in seconds and *Y*-axis shows the 340/380 (bound/unbound calcium) ratio, in individual neurons depicted by different colours.

To determine whether the delayed calcium increase in the presence of 1 µM myrcene was due to calcium influx, or release from calcium stores, the experiment was repeated in the same neurons demonstrating terpene mediated inhibition of capsaicin responses. Following washout of medium and 45 min recovery, terpene application in calcium magnesium free HHBSS and 200 µM EGTA (pH 7.4), also resulted in delayed capsaicin responses (Figure 4a), indicating that the delayed rise in calcium was due to release from intracellular stores, and not due to calcium influx. The delayed response was eliminated after pre-treatment with the smooth endoplasmic reticulum calcium (SERCA) pump inhibitor thapsigargin (3 µM) in calcium magnesium free HHBSS containing EGTA (pH 7.4) (Figure 4b). This finding indicates that the delayed capsaicin responses were due to calcium release from the endoplasmic reticulum, while calcium influx was completely blocked in the presence of terpenes in most capsaicin sensitive neurons.

#### 2.1.5. Terpene Mediated Capsaicin Response Inhibition Does Not Involve CB_1_ or CB_2_ Receptors

**Figure 5:**
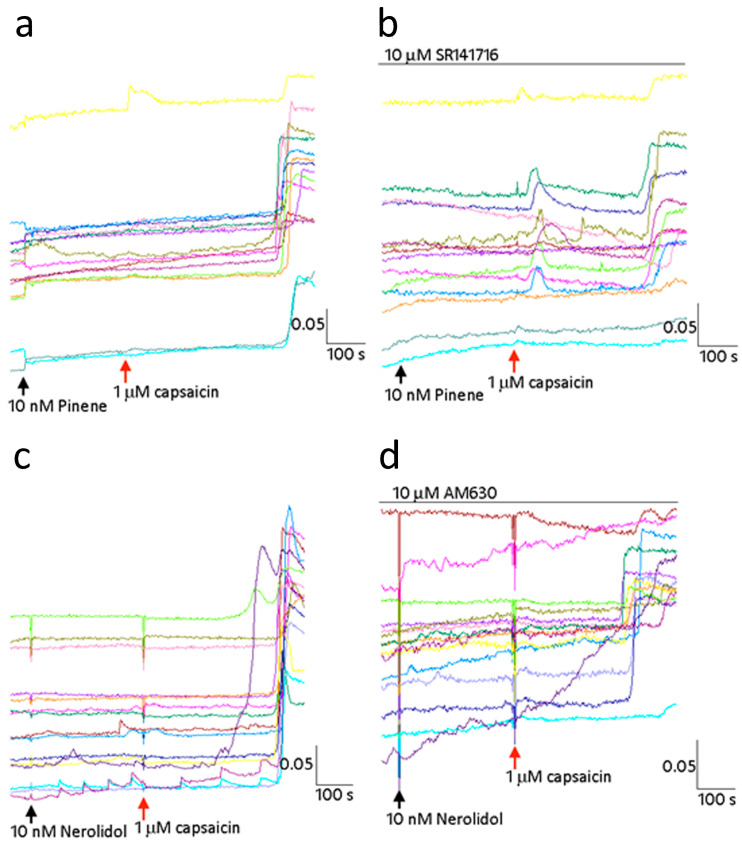
Terpene inhibition of TRPV1 is not sensitive to CB_1_ or CB_2_ receptor antagonists. Sample traces showing capsaicin response inhibition in the presence of 10 nM pinene (**a**) is not completely reversed by the CB_1_ receptor antagonist (**b**). Traces showing capsaicin response inhibition due to 10 nM nerolidol (**c**) is unaffected by the presence of the CB_2_ receptor antagonist AM630 (**d**). Scale bars show time in seconds on the *X* axis and the 340/380 ratio on the *Y* axis.

Neurons demonstrating delayed capsaicin responses in the presence of terpenes, had a change of medium and rest period of 45 min to overcome desensitization due to capsaicin application. Further application of terpineol, nerolidol, myrcene, or caryophyllene in the presence of the CB_1_ receptor antagonist SR141716A or the CB_2_ antagonist AM630, failed to restore calcium influx in response to capsaicin (Figure 5a–d).

#### 2.1.6. Mechanism of Terpene Mediated TRPV1 Inhibition

**Figure 6:**
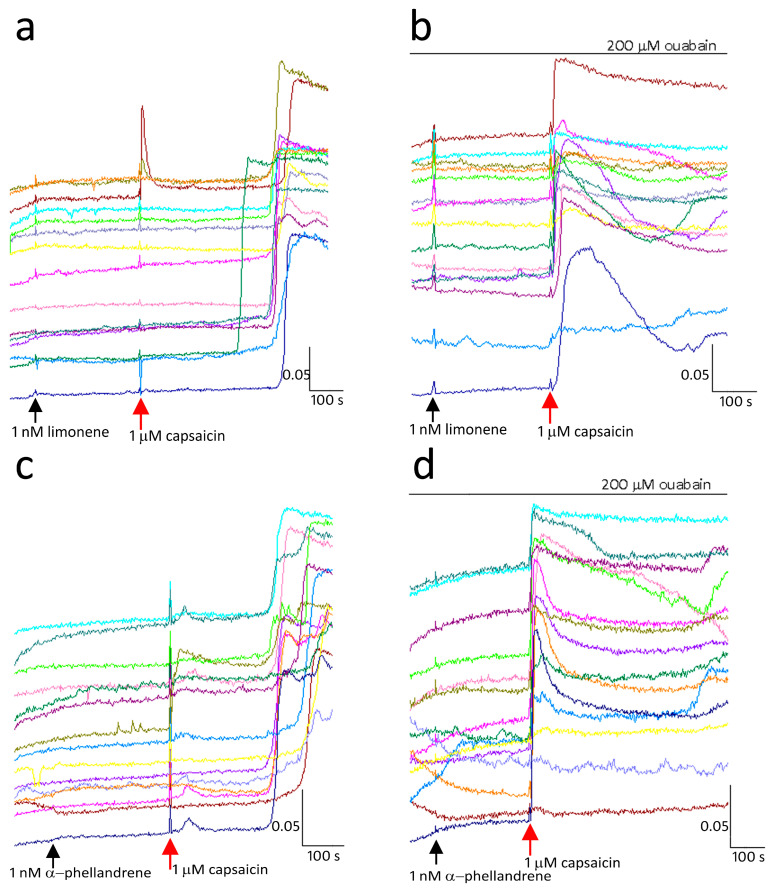
TRPV1 inhibition by terpenes is ouabain-sensitive. Sample traces from individual neurons showing capsaicin response inhibition by 1 nM limonene (**a**) was reversed in the presence of the Na^+^/K^+^ ATPase inhibitor ouabain (**b**). Similarly, TRPV1 inhibition by 1 nM α-phellandrene (**c**) was also reversed in the presence of ouabain (**d**). Scale bar: *X*-axis shows the time in seconds. *Y*-axis shows the intracellular 340/380 (bound/unbound calcium) ratio, in individual neurons depicted by different colours.

Normal latency, i.e., immediate calcium influx in response to capsaicin, was restored in the presence of the Na^+^/K^+^ ATPase inhibitor ouabain. Capsaicin response inhibition due to 0.001 µM limonene (Figure 6a), was reversed 45 min after medium washout, in the presence of 200 µM ouabain (Figure 6b). Similarly, capsaicin responses inhibited by 0.001 µM α phellandrene (Figure 6c), were restored in the presence of 200 µM ouabain (Figure 6d).

**Figure 7:**
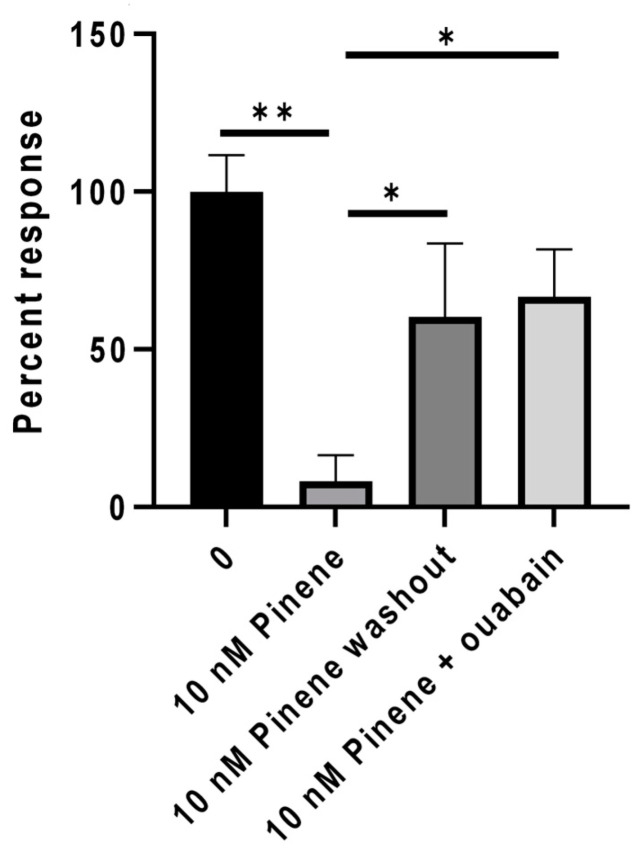
Graph showing control capsaicin responses in DRG neurons (n = 6), which were significantly reduced in the presence of 10 nM pinene (** *p* < 0.01, n = 6). Following washout, capsaicin responses were restored in the absence of pinene (* *p <* 0.05, n = 3). Addition of pinene in the presence of 500 µM ouabain significantly reversed pinene induced response inhibition (* *p* =< 0.05, n = 3). Data shown as mean ± SEM using the Mann–Whitney test in Graphpad Prism.

Capsaicin response inhibition by α-pinene (Figure 7), α-terpineol, α-phellandrene, nerolidol, borneol, and β-caryophyllene was also reversed in the presence of the Na^+^/K^+^ ATPase inhibitor ouabain (200–500 µM).

### 2.2. Immunofluorescence of α1Na^+^/K^+^ ATPase and TRPV1

**Figure 8:**
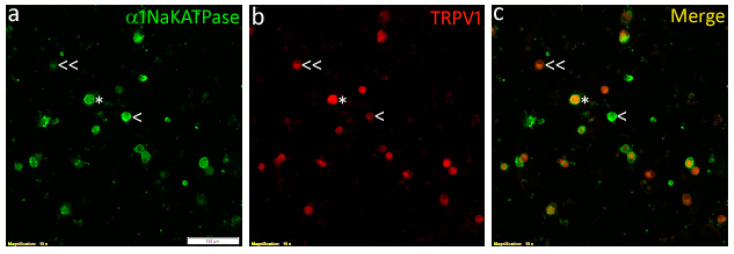
Widefield immunofluorescence images of rat cultured DRG neurons showing neurons positive for expression of α1Na^+^/K^+^ ATPase (green, (**a**)), TRPV1 (red, (**b**)), and the merged image showing co-expression (yellow, (**c**)). Examples of neurons showing co-expression are indicated by asterisk (*), Na^+^/K^+^ ATPase positive/TRPV1 negative are indicated by single arrowhead (<), and Na^+^/K^+^ ATPase negative/TRPV1 positive indicated by double arrowhead (<<). Scale bar in ‘a’ represents 100 µm.

In order to determine the co-expression of the capsaicin receptor TRPV1 and the ouabain sensitive α1Na^+^/K^+^ ATPase, we double immunostained DRG neurons for these targets. The findings showed that some neurons were positive for either α1Na^+^/K^+^ ATPase, or TRPV1 alone, while others showed co-localization of both (Figure 8a–c).

**Figure 9:**
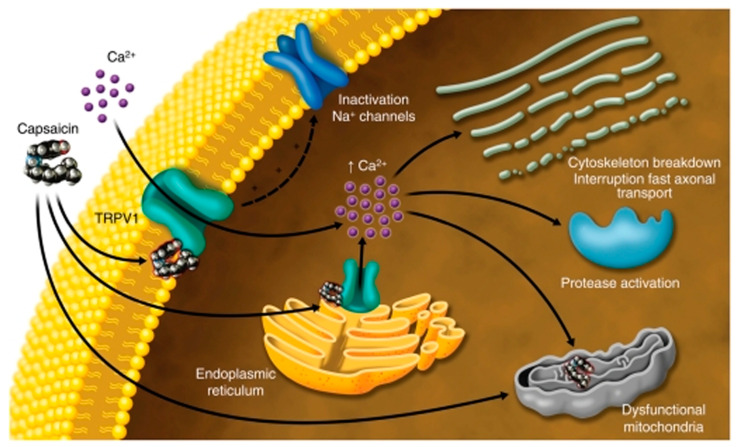
Image depicting capsaicin activation of TRPV1 receptors in the plasma membrane eliciting calcium influx, while activation of endoplasmic reticulum bound TRPV1 receptors elicits release of stored calcium into the cytosol. The effect of terpenes in our study was to block activation of the plasma membrane bound TRPV1 receptors, while the ER bound TRPV1 receptors were unaffected, giving rise to the delayed capsaicin response. Restoration of calcium influx in the presence of the Na^+^/K^+^ ATPase inhibitor ouabain indicates that terpenes activate the Na^+^/K^+^ ATPase, potentially resulting in hyperpolarization. Image from our publication; see Ref. [33].

The mechanisms underlying the observations in this study are schematically depicted in Figure 9. Plasma membrane bound TRPV1 receptors permit calcium influx when activated by capsaicin, and calcium release from the endoplasmic reticulum. The presence of terpenes prevents calcium influx in response to capsaicin but permits calcium release from the endoplasmic reticulum.

## 3. Discussion

The results of our study show that terpenes ranging in concentration from 0.001 to 100 µM have potent inhibitory effects in DRG neurons, by blocking calcium influx in response to capsaicin used for activating TRPV1. The majority of capsaicin sensitive neurons showed complete insensitivity to capsaicin for several minutes before increased calcium was observed. These delayed capsaicin responses were maintained in calcium/magnesium free HHBSS-EGTA but were abolished in the presence of the smooth endoplasmic reticulum calcium (SERCA) pump inhibitor, thapsigargin, in calcium magnesium free HHBSS -EGTA. Thus, the delayed capsaicin responses were due to calcium released from internal stores, observed between 6 and 8 min after applying capsaicin, while extracellular calcium influx was completely abolished during this time. Inhibition of calcium influx in response to capsaicin was observed in the presence of all the terpenes tested here, and at all concentrations from 0.001 to 100 micromolar, which affected up to 100% capsaicin sensitive neurons in some experiments, indicating potent inhibition of TRPV1 by terpenes. There was no dose related pattern of TRPV1 inhibition, and while more than 75% of capsaicin sensitive neurons were completely inhibited, the duration of calcium influx inhibition due to terpenes is not known.

TRPV1 inhibition by the terpenes was reversible, as capsaicin responses were restored 45 min after washout of medium and in the absence of the terpenes. Terpene mediated TRPV1 inhibition did not involve the cannabinoid receptors CB_1_ or CB_2_, as both the CB_1_ receptor antagonist SR141716 and the CB_2_ receptor antagonist AM630 did not change the inhibitory effects of the terpenes. Thus, terpenes appear to mediate their TRPV1 inhibitory effects independently of the cannabinoid receptors.

The major finding of this study is that terpene inhibition of TRPV1 was reversed in the presence of the sodium potassium (Na^+^/K^+^ ATPase) pump inhibitor ouabain, indicating that the terpenes activated the Na^+^/K^+^ ATPase, an intrinsic enzyme in the plasma membrane, which is responsible for the coupled active transport of Na^+^ and K^+^ in most eukaryotic cells.

Na^+^/K^+^ ATPase is a plasma membrane bound enzyme that transports three Na^+^ out of the cell and two K^+^ into the cell, playing a critical role in maintaining the Na^+^ and K^+^ gradient across the plasma membrane. This gradient is essential for maintaining the resting membrane potential and excitable properties of neurons [34,35,36]. Physiological activation of the sodium pump follows elevated intracellular sodium levels, due to action potential generation. Thus, terpene activation of the Na^+^/K^+^ ATPase had a similar effect, potentially leading to hyperpolarization and neuronal inhibition, which is likely to affect other receptors such as TRPA1 and TRPM8 as well. This will have to be tested in further studies. Experiments using calcium magnesium free extracellular medium and thapsigargin pretreatment showed that the delayed capsaicin responses represented the elevated intracellular calcium due to calcium release from intracellular stores. This observation further shows selective activation of the plasma membrane bound Na^+^/K^+^ ATPase by the terpenes, which blocked calcium influx, while the intracellular Ca^2+^ATPase was unaffected, permitting store operated calcium release.

Na^+^/K^+^ ATPase is a heterodimer of α and β subunits [37], and the mRNAs for Na^+^/K^+^ ATPase α1, α3, and β1 subunits are found in DRG neurons [34,38,39,40]. The α1 subunit is expressed in both small- and large-diameter DRG neurons, while the α3 subunit is mainly distributed in large neurons [41,42], and the α1β1 and α3β1 isozymes are both expressed in adult rat DRG neurons [38,39,41]. The functional Na^+^/K^+^ pump is a heterodimer of different isozymes α1β1 and α3β1 with differing sensitivity to ouabain [34,42]. Individual neurons express different ratios of α1β1 and α3β1 subtypes, and our results reflect this heterogeneity. The membrane current produced by Na^+^/K^+^ ATPase activity in DRG neurons is primarily mediated by α1 Na^+^/K^+^ ATPase and is ouabain sensitive with maximal inhibition at 1 mM ouabain [34,39,42].

The ouabain concentrations used in our study were based on electrophysiological studies [34,42], which showed the dose related inhibition of the Na^+^/K^+^ pump current by ouabain, in small diameter DRG neurons. These studies showed that the maximal inhibition of the Na^+^/K^+^ pump by ouabain was at 1 mM, being related to the contribution of two isoenzymes of the pump with differing sensitivities to ouabain. The ouabain concentrations used in our study, i.e., 200 µMol and 500 µMol, were far less (20% and 50%, respectively), than the concentration required for maximal inhibition, and chosen to avoid off target effects. In another study, the endogenous follistatin-like 1 (FSTL1) was shown to inhibit sensory transmission by activating the Na^+^/K^+^ ATPase in DRG neurons [40]. This study used 100 µMol ouabain due to the low sensitivity of the α1 subunit, which binds ouabain at 100 µMol but not at 1 µMol [34,42].

Our results indicate that terpenes have similar effects of inhibiting neuronal activation via an ouabain sensitive mechanism, by activating Na^+^/K^+^ ATPase. The membrane potential effects of terpenes will be confirmed in future studies. While most capsaicin sensitive neurons showed complete inhibition in the presence of terpenes, others showed reduced responses, possibly reflecting varying effects at the different isozymes.

Although Na^+^/K^+^ ATPase is ubiquitous, in situ hybridization and immunostaining showed that the α1 subunit was expressed in ~51% of rodent DRG neurons, and in ~63% of small neurons [39]. Another study described the expression of the α1 subunit containing Na^+^/K^+^ ATPase by up to 80% DRG neurons, especially in the small diameter nociceptive pain and temperature sensing neurons [40]. Our immunofluorescence data in the present study also demonstrates co-localization of the α1 Na^+^/K^+^ ATPase with TRPV1 expressing DRG neurons, highlighting the plasma membrane Na^+^/K^+^ ATPase as an important target for the modulation of pain. Na^+^/K^+^ ATPase activity and neuronal survival in chick DRG depends on NGF [43], highlighting its role in pain signalling. Further studies to assay Na^+^/K^+^ ATPase activation by terpenes should be useful in providing an estimate of their potency and efficacy. Terpene activation of Na^+^/K^+^ ATPase has not been described before, and this effect is likely to have additive or synergistic effects with cannabinoids acting via CB_1_ and CB_2_ receptors. The immunofluorescence of α1 Na^+^/K^+^ ATPase and TRPV1 in our study showed co-expression in some neurons, while others were positive for one but not the other. It is likely that the maximum inhibition due to terpenes was observed in those neurons that expressed both targets, reflecting the heterogeneous expression of receptors and different isoforms of the sodium pump in DRG neurons. Neurons positive for TRPV1 but α1β1 Na^+^/K^+^ ATPase negative, would not be expected to show terpene mediated inhibition, and would show normal responses to capsaicin. Some capsaicin sensitive neurons showed 100% inhibition by terpenes, while others did not. As the capsaicin receptor TRPV1 is expressed by a large proportion of DRG neurons [21], terpene inhibition of capsaicin responses likely reflects co-expression of TRPV1 and the ouabain sensitive α1β1 Na^+^/K^+^ isozyme, which is not expressed in all neurons.

Terpenes may share a common effect of mediating hyperpolarization with synthetic cannabinoids, via G-protein gated inward rectifier potassium (GIRK) channel activation [44,45]. A similar mechanism of action was described for the Nociceptin/OFQ inhibition of capsaicin responses in nodose ganglion neurons, which was reversed by the GIRK channel inhibitor Tertiapin [28]. Further studies are required to quantify terpene mediated effects on membrane potential and their sensitivity to ouabain in DRG neurons, as terpene treatment failed to show CB_1_ or CB_2_ receptor mediated changes in membrane potential using AtT20 Flpin cells transfected with the human cannabinoid receptors [46].

The sodium channels Nav1.7, 1.8, and 1.9 play an important role in pain signalling and are upregulated in clinical conditions of inflammation [47,48,49]. Inflammation in rat DRG neurons is also associated with upregulation of Nav1.7, accompanied by a compensatory upregulation of both α1 and α3 subunits of the Na^+^/K^+^ ATPase to counter the increased sodium influx for maintaining osmotic homeostasis. Further, the blockade of the Na^+^/K^+^ ATPase with ouabain, stimulated neuronal firing and resulted in neuronal lysis, showing that Na^+^/K^+^ ATPase regulation during major inflammatory disease states is critical for homeostatic protection of primary afferent neurons [50]. Thus, terpene activation of the Na^+^/K^+^ ATPase may provide an alternative strategy to regulate neuronal activation, for developing novel pain therapy.

In conclusion, our findings suggest that terpenes are potent inhibitors of pain signalling, via ouabain sensitive Na^+^/K^+^ ATPase activation. Combinations of terpenes and the cannabinoids THC, CBD, and CBG are likely to show enhanced effects, via additive or synergistic effects involving different signalling pathways. Our in vitro model would be particularly useful for developing topical analgesic treatments as it shows their direct effect on cutaneous nociceptors.

## 4. Materials and Methods

DRG neurons were prepared as described earlier [2], using adult female Wistar rats (Charles River, Harlow, UK), following approved procedures (by the Animal Welfare Ethical Review Body, Imperial College London, and in keeping with the 3Rs ARRIVE guidelines). Bilateral DRG from all levels were harvested in Ham’s F12 medium containing penicillin/streptomycin (100 µg/mL each), and enzyme digested in 2 mL Ham’s F12 medium containing antibiotics, collagenase (0.2%) and dispase (0.5%), at 37 °C for 3 h. The enzyme digested tissue was mechanically dissociated by pipetting in 1 mL BSF2 medium containing 100 ng/mL NGF and 50 ng/mL GDNF, soybean trypsin inhibitor and DNase, to obtain a cell suspension containing 150,000–200,000 neurons. This was diluted in BSF2 medium (containing NGF and GDNF), and 200 µL cell suspension containing 8000–10,000 neurons plated on each of 20 glass-bottom petri dishes (MatTek Corp., Ashland, MA, USA), precoated with 20 μg/mL each of poly-l-lysine and laminin. The culture dishes were incubated at 37 °C for 45 min to allow the cells to attach before adding 2 mL BSF2 medium containing NGF (100 ng/mL) and GDNF (50 ng/mL) to each dish. Then, 24 h later, 5 µM cytosine arabinoside was added to all dishes to inhibit the growth of non-neuronal cells.

### 4.1. Calcium Imaging

Calcium imaging was performed between 2 and 4 days after plating the neurons. The cultures were rinsed with warm 1 mL HEPES buffered HBSS (HHBSS) containing 10 mM HEPES and 0.1% BSA (pH 7.4). Then, 1 mL HHBSS containing 2 µMol Fura2AM (Life Technologies, Paisley, UK), was added to each dish, and the petri dishes were incubated at 37 °C for 40 min in the dark. The medium was then replaced with HHBSS for 20 min to allow de-esterification of the Fura2AM. Fresh 2 mL HHBSS was added to each dish for each experiment. A total of 12–15 healthy neurons were selected using a 10× objective lens under brightfield illumination for each experiment, and a region of interest was highlighted in each neuron (Figure 1a). The neuron culture was alternately excited at 340 and 380 nm (λex) (510 λem) wavelengths to obtain the intracellular bound/unbound Ca^2+^ ratio. A stable baseline of the 340/380 nm ratio was recorded, and 0.1% ethanol (vehicle) was applied, followed by 1 µM capsaicin after 5 min. One image was captured every two seconds in each of 3 channels: brightfield, 340 nm and 380 nm λex, as previously described [21], and the mean 340/380 nm λex ratio was recorded for each neuron to reveal intracellular Ca^2+^ changes due to terpene or capsaicin application. In each experiment the largest calcium responses were selected for analysis, with a minimum change of 0.02 from the baseline. Responses were recorded as the difference between baseline (mean 340/380 nm λex ratio) just before addition of the terpene or capsaicin, and peak after the addition and expressed as a percentage of the vehicle treated control average. In separate dishes, following baseline recording, individual terpenes were added at either 0.001, 0.01, 0.1, 1, 10 or 100 μM, followed 5 min later by 1 μM capsaicin (Figure 1a). Effects of CB_1_ or CB_2_ antagonists and ouabain were evaluated in the same neurons demonstrating inhibition by washout of medium followed 45 min later by adding the antagonist prior to adding the terpenes.

The terpenes Borneol, Phytol, Terpenolene, α-pinene, (R)-(+)-Limonene, α-phellandrene, myrcene, β-caryophyllene, nerolidol, and α-terpineol were freshly prepared prior to use at 1000× final concentration in ethanol. Stocks of CB_1_ antagonist Rimonabant (SR141716 10 mM), CB_2_ antagonist AM630 (10 mM), thapsigargin (3 mM, Cayman chemical, Ann Arbor, MI, USA), Ouabain (100 mM, Abcam Ltd., Cambridge, UK) were prepared in DMSO, aliquoted and stored at −20 °C, until use. Aliquots of capsaicin stock solution (100 mM) prepared in ethanol were frozen at −20 °C and used for preparing intermediate stock solution of 500 µM prior to use. All chemicals were obtained from Merck (Gillingham, UK), unless indicated otherwise.

### 4.2. Data Analysis

Capsaicin responses from 12 to 15 neurons were averaged for each concentration of each terpene per rat and normalized to vehicle treated controls. Capsaicin responses were assessed for latency (time taken to respond from applying capsaicin), and amplitude of response (peak minus baseline) at the point of capsaicin application, and averages were normalised to vehicle treated controls. Average values for each cannabinoid concentration were compared to the control using the Mann–Whitney test in GraphPad Prism (v10) software. Here, * *p* < 0.05 was considered statistically significant.

### 4.3. Immunostaining

A total of 48 h after plating, rat DRG neuron cultures were fixed in 2% paraformaldehyde for 15 min and rinsed 3 × 5 min in PBS containing 0.01% sodium azide. Following permeabilization with chilled methanol for 3 min, neurons were incubated with primary antibodies mouse monoclonal anti α1Na^+^/K^+^ ATPase (Abcam, #AB7671-1001, 1:250), and rabbit anti-TRPV1 (GlaxoSmithKline, Harlow, Essex, UK, 1:500), for 1 h at room temperature. Following 3 × 5-min rinses with PBS containing 0.01% sodium azide, secondary antibodies goat anti mouse (Life Technologies, F(ab)’2 Alexafluor Plus 488 #A48286, 1:200) and goat anti rabbit (Life Technologies, Alexa fluor 546, #A11010, 1:200), in antibody diluent containing 40% foetal calf serum were applied for 1 h at room temperature. The glass bottom coverslips were detached with proprietary glass removal fluid (MatTek #PDCF OS 30), rinsed thrice in PBS containing sodium azide, and mounted on glass slides (superfrost) in glycerol containing antifade agent (DABCO). Coverslip edges were sealed with nail varnish. Multichannel images were acquired using epifluorescence optics on a BX43 Olympus microscope with UV illumination and Olympus Cellsens software (cellSens Dimension 1.7, Olympus, Tokyo, Japan).

## 5. Conclusions

In conclusion, our findings show that terpenes are potent inhibitors of pain signalling via ouabain sensitive Na^+^/K^+^ ATPase activation.

## Figures and Tables

**Table 1:** Percentage capsaicin sensitive neurons (mean ± SEM), showing inhibition of calcium influx in the presence of different terpenes (data from n = 3–4 rats/terpene).

Borneol	Terpineol	α-pinene	Nerolidol	β caryophyllene
37.6 ± 19.3	77.1 ± 6.9	79.7 ± 5.6	75.5 ± 14.4	62.7 ± 19
Terpinolene	Myrcene	Limonene	α phellandrene	Phytol
64.5 ± 7.3	71.1 ± 11.9	40.9 ± 4	62 ± 14	62.7 ± 4.6

## Data Availability

Data have been included in the manuscript.
